# Supplementary material for: Whi7 is an unstable cell-cycle repressor of the Start transcriptional program
Source: Nat Commun. 2017 Aug 24;8:329. doi: 10.1038/s41467-017-00374-1 (PMC5571219; doi:10.1038/s41467-017-00374-1)
Supplement: Supplementary file 1 — Supplementary Information [file 41467_2017_374_MOESM1_ESM.pdf]

Title: Supplementary Information
Description: Supplementary Figures and Supplementary Table

Title: Peer Review File
Description:

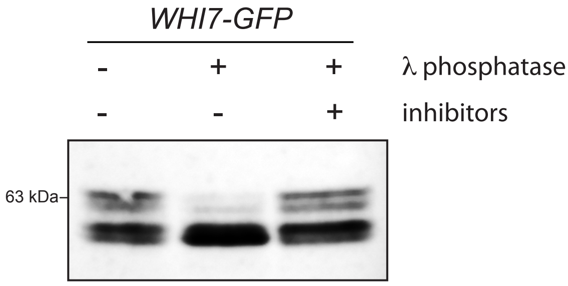

Supplementary Figure 1. Analysis of Whi7 phosphorylation by λ phosphatase assay. Cell extracts from the *WHI7-GFP* strain (JCY1802) were incubated with λ protein phosphatase in the absence or presence of phosphatase inhibitors. Electrophoretic migration of Whi7 was analyzed by western blot.

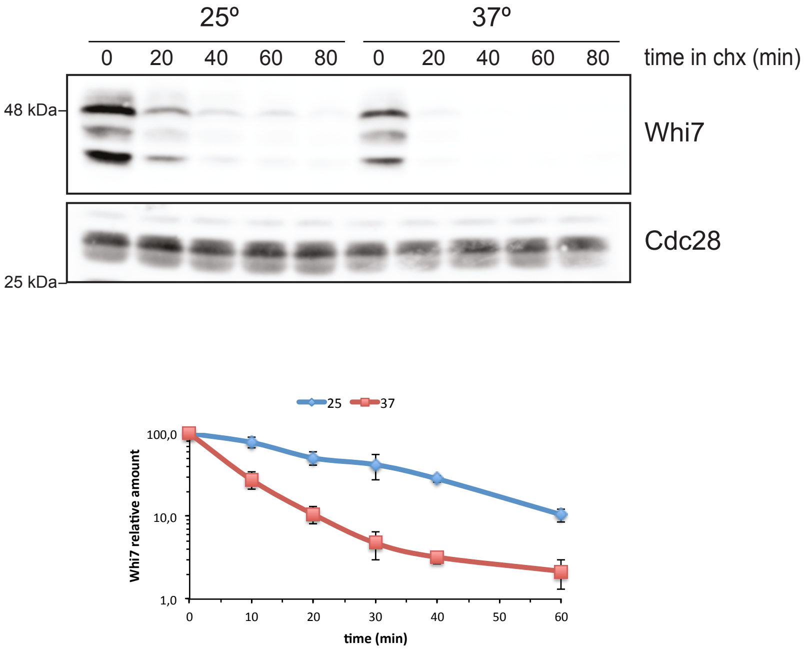

Supplementary Figure 2. Analysis of Whi7 stability at different temperatures. Exponentially growing cultures of the *WHI7-HA* (JCY1728) strain were split, incubated for 3 hours at 25°C or 37°C and then 100 µg mL^{-1} cycloheximide was added. Whi7 protein level was analyzed at the indicated time after the addition of cycloheximide by western blot. Cdc28 is shown as loading control. Graph represents the relative amount of Whi7 protein related to Cdc28. Values are the mean and s.d. derived from three experiments.

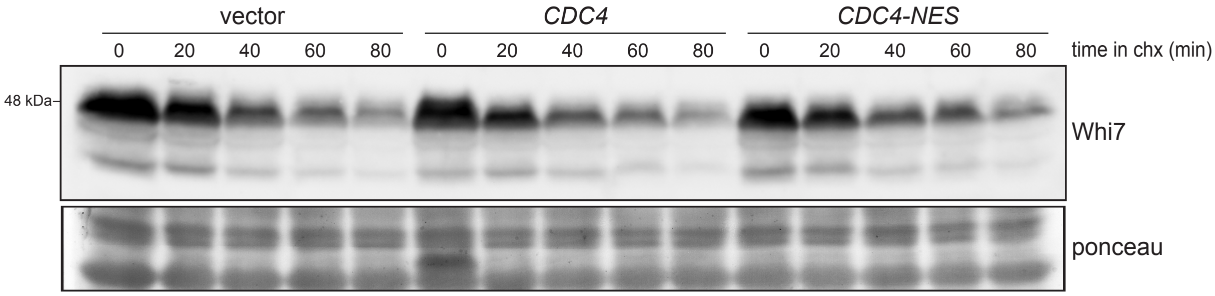

Supplementary Figure 3. Effect of forced nuclear export of Cdc4 in Whi7 protein stability. Exponentially growing cells of the *grr1 WHI7-HA* (JCY1760) transformed with an empty vector and a centromeric plasmid expressing wild type Cdc4 or Cdc4 fused to a nuclear export sequence (NES), were incubated in the presence of 100 μg mL^{-1} cycloheximide. Whi7 protein level was analyzed at the indicated time after the addition of cycloheximide by western blot. Ponceau staining is shown as loading control.

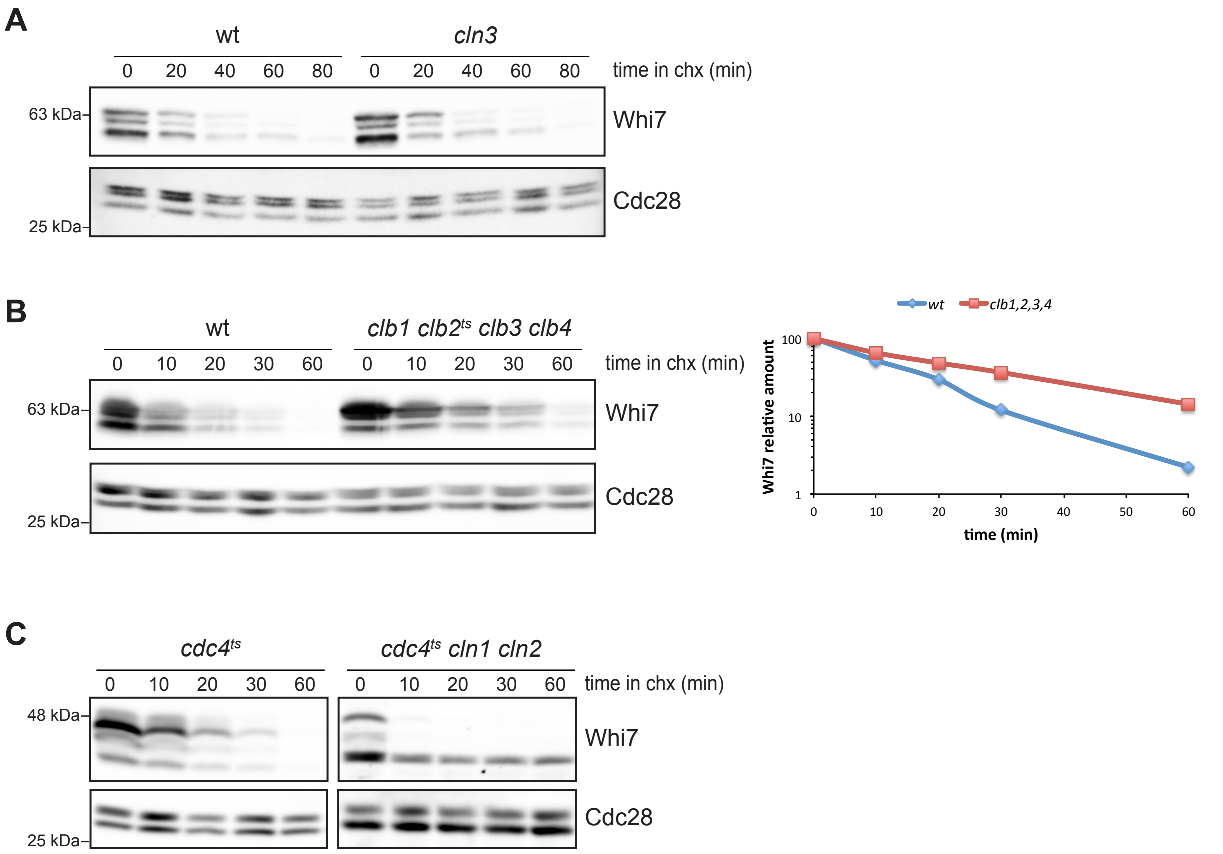

Supplementary Figure 4. Analysis of Whi7 degradation in a cyclin mutant strains.

A) Exponentially growing cells of *WHI7-GFP* (JCY1746) and *cln3 WHI7-GFP* (JCY1815) strains were incubated in the presence of 100 µg mL^{-1} cycloheximide. Whi7 protein level was analyzed at the indicated time after the addition of cycloheximide by western blot. Cdc28 is shown as loading control. B) Whi7 protein stability in exponentially growing cells of *WHI7-GFP* (JCY1746) and *clb1 clb2^{ts} clb3 clb4 WHI7-GFP* (JCY1991) strains incubated at 37°C for 3 hours, was assayed as described in A. C) Whi7 protein stability in exponentially growing cells of *cdc4^{ts} WHI7-HA* (JCY1757) and *cdc4^{ts} cln1 cln2 WHI7-HA* (JCY2013) strains incubated at 37°C for 3 hours, was assayed as described in A.

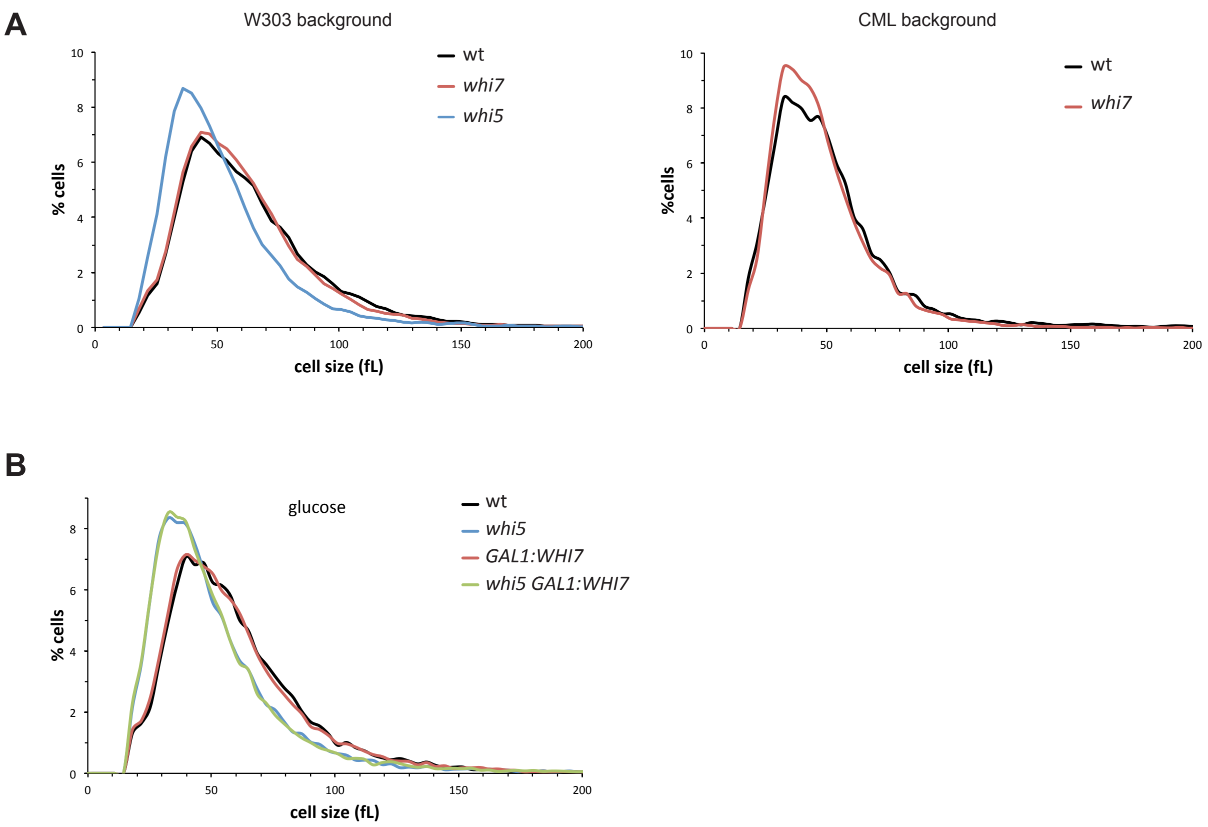

Supplementary Figure 5. Analysis of the cell size in *whi7* mutant strains. *A) Left panel:* cell size distribution in exponentially growing cultures of the wild type (W303), *whi7* (JCY1819) and *whi5* (JCY1874) strains. *Right panel:* cell size distribution in exponentially growing cultures of the wild type (CML128) and its derivative *whi7* strain used in Yahya et al. 2014. *B)* Cell size distribution in exponentially growing cultures on YPD medium (*GAL1* promoter repressed to mimic *whi7* mutation) of the wild type (W303)*, whi5* (JCY1874), *GAL1:WHI7* (JCY1804) and *whi5 GAL1:WHI7* (JCY1885), strains*.* Graphics are the moving average from at least six independent cultures.

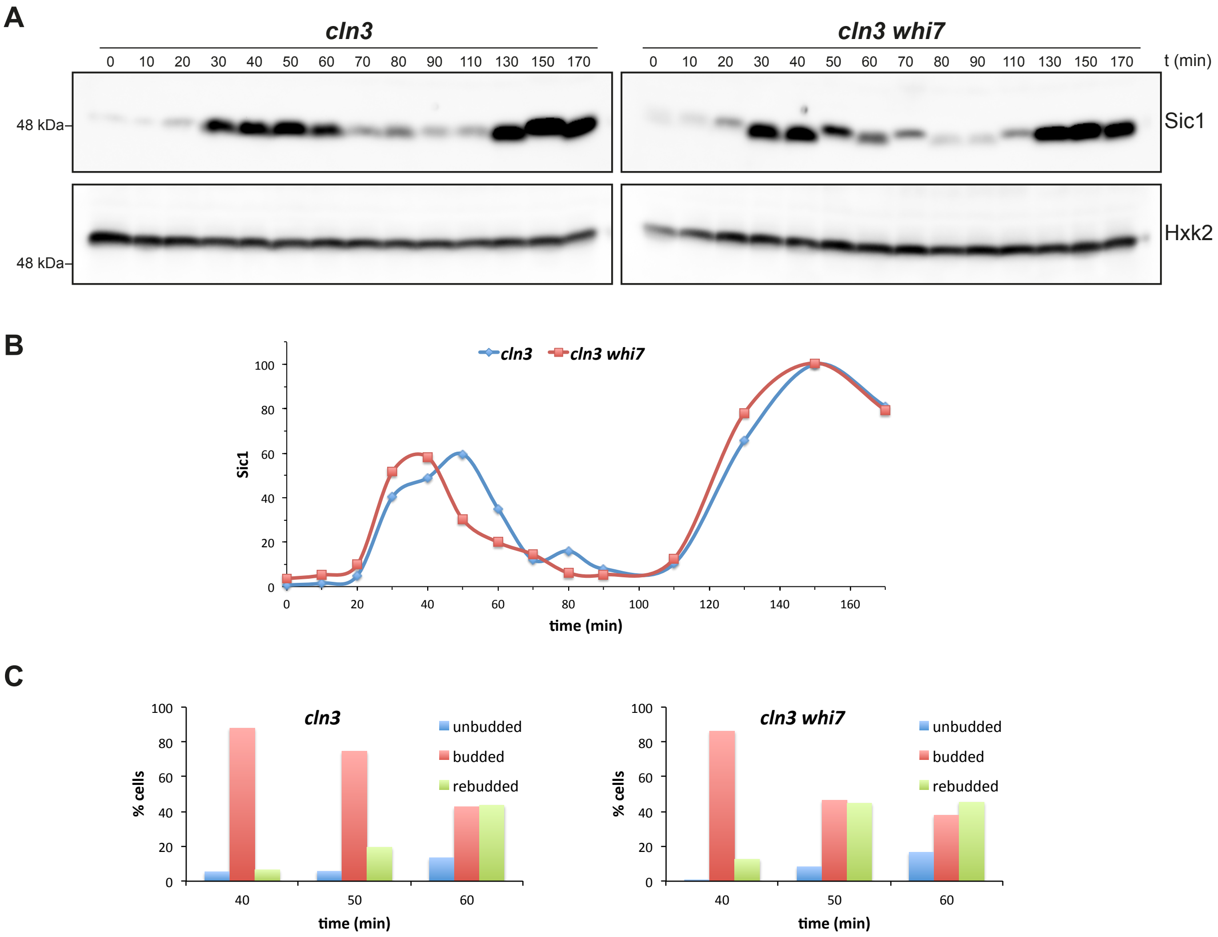

Supplementary Figure 6. Analysis of the cell cell cycle progression in *cln3 whi7* mutant strain. *A)* Cultures of *cdc15 cln3 SIC1-myc* (JCY1837) and its derivative *whi7* (JCY1896) mutant strain were arrested in telophase by incubation at 37°C. After 3 hours, cells were transferred to 25°C and cell cycle progression was analyzed at the indicated time. Western blot shows Sic1 and Hxk2 (as loading control) protein level. *B)* Plot represents the relative amount of Sic1 protein relative to Hxk2. *C)* Budding as a marker of cell cycle initiation was monitored at the indicated times. Note that *cdc15* mutant often shows after release from the arrest a delay in cell separation giving rise to the apparition of rebudded cells.

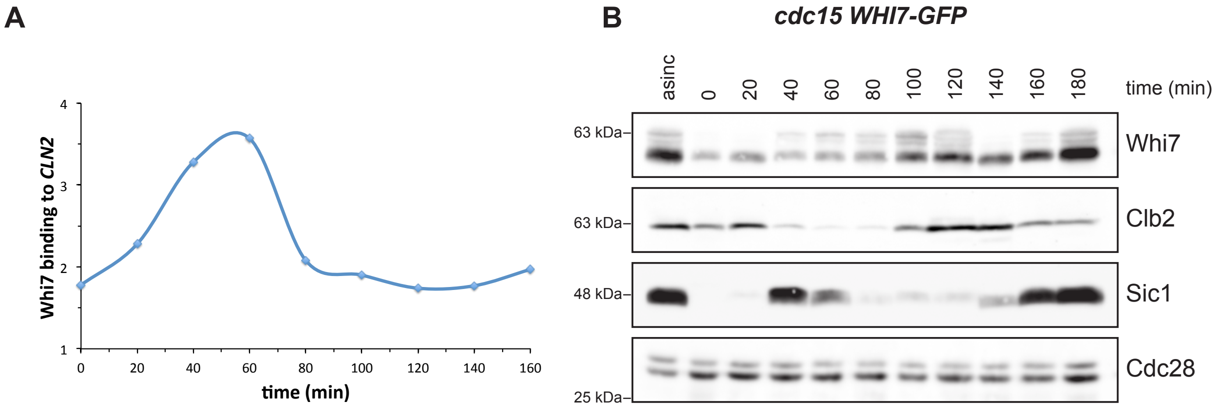

Supplementary Figure 7. Analysis of Whi7 association with *CLN2* promoter. Exponentially growing cultures of *cdc15 WHI7-GFP* (JCY1802) strain were arrested in telophase by incubation for 3 hours at 37°C. After release from the arrest, Whi7 binding to *CLN2* promoter was investigated by ChIP assays. Values represent the enrichment in purified fractions of *CLN2* specific promoter fragments respect to intergenic DNA relative to the no tag control strain. Western blot show Whi7, Clb2, Sic1, and Cdc28 (as loading control). Cell cycle progression was monitored by the oscillations of Clb2, Sic1 and bud emergence that indicated execution of Start at 60-80 min. samples.

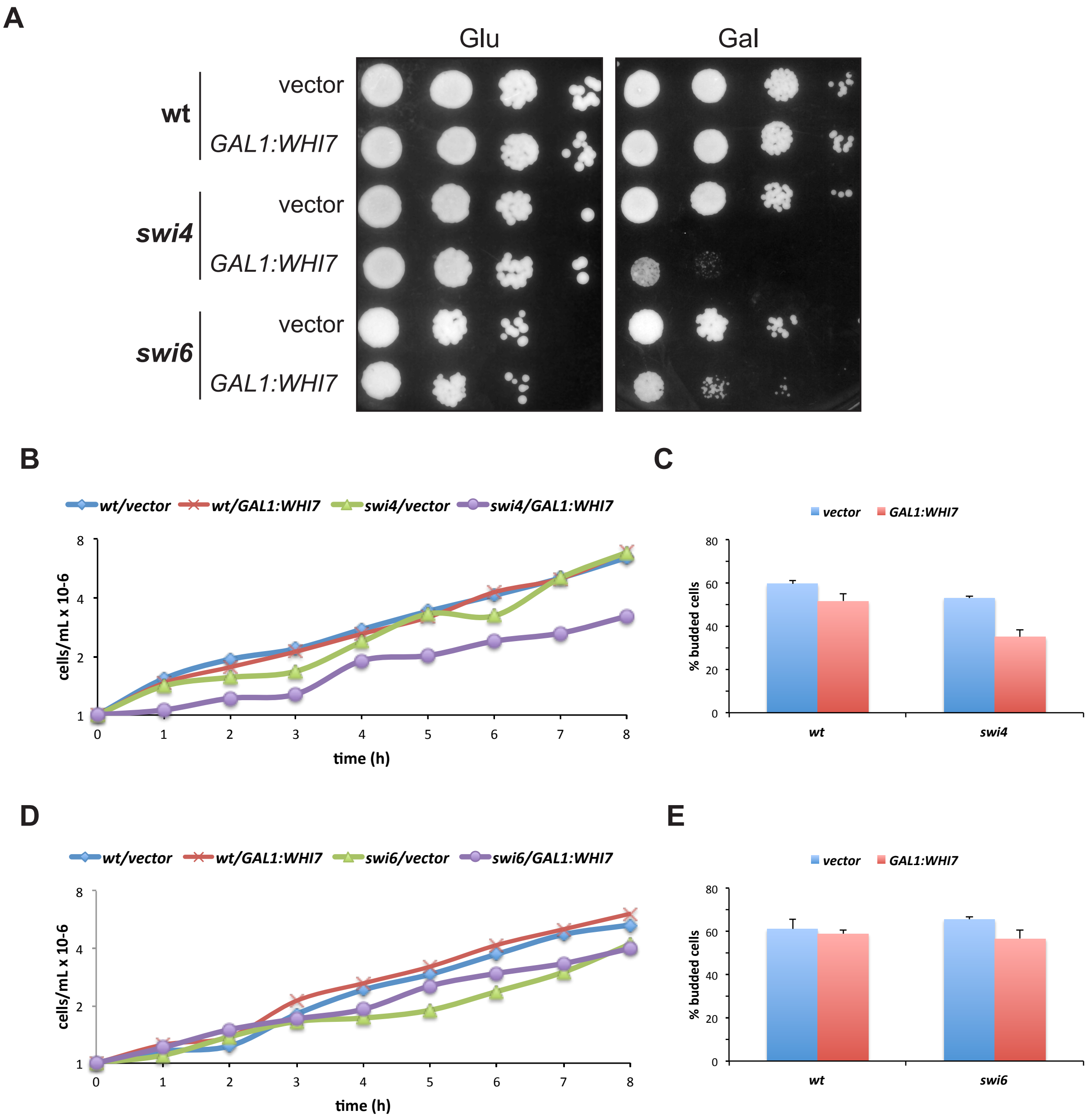

Supplementary Figure 8. Effect of *WHI7* overexpression in cell cycle progression in *swi4 and swi6* mutant strains.

A) 10-fold serial dilutions from exponentially growing cultures of the wild type (W303), *swi4* (JCY167) and *swi6* (JCY325) mutant strains transformed with an empty vector or a centromeric plasmid expressing *WHI7* under the control of the *GAL1* promoter (pGAL1:WHI7) were spotted onto SC-Glu and SC-Gal medium and incubated at 25°C for 3 days. *B,D)* The same strains were grown overnight on SC-Raf. Galactose to 2% was added to induce overexpression of *WHI7* and the increase in cell number was analysed at the indicated times. *C,E)* Cell cycle distribution of cells after 3 hours from the addition of galactose. Plot represents percentage of budded cells (mean and s.d.) derived from three independent cultures.

Figure 1B

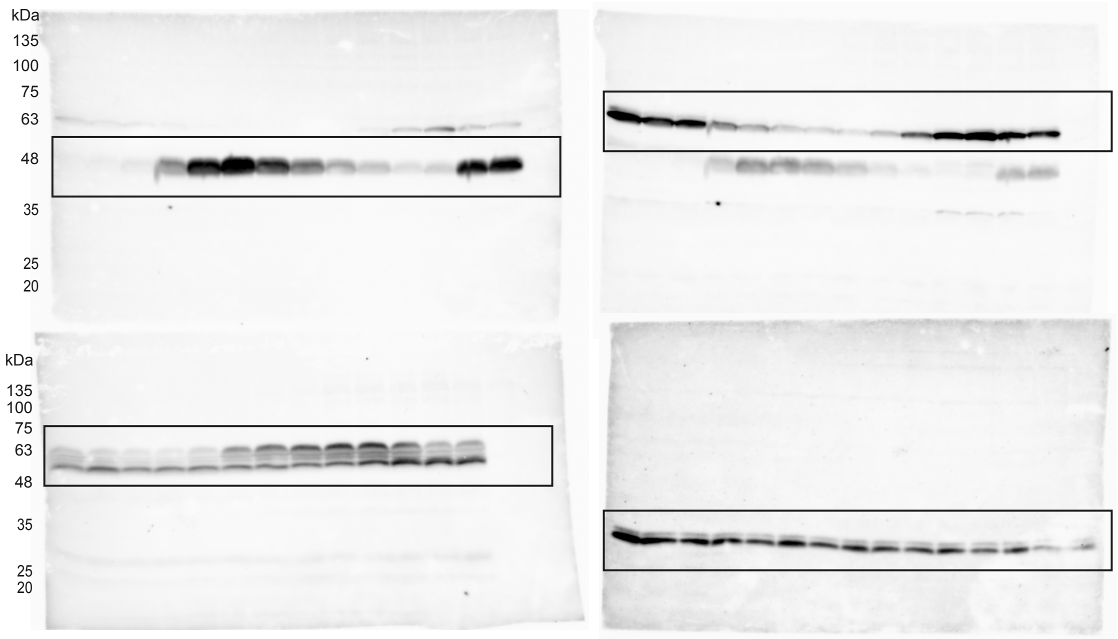

Supplementary Figure 9. Uncropped western blots shown in Figure 1

Figure 2A

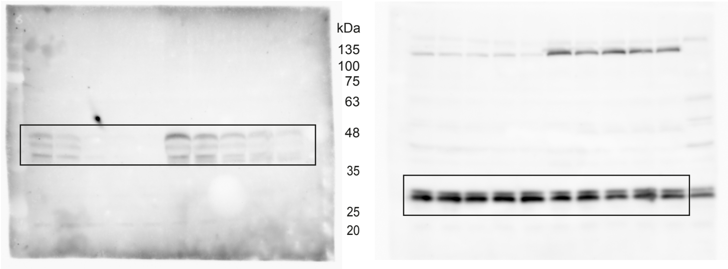

Figure 2B

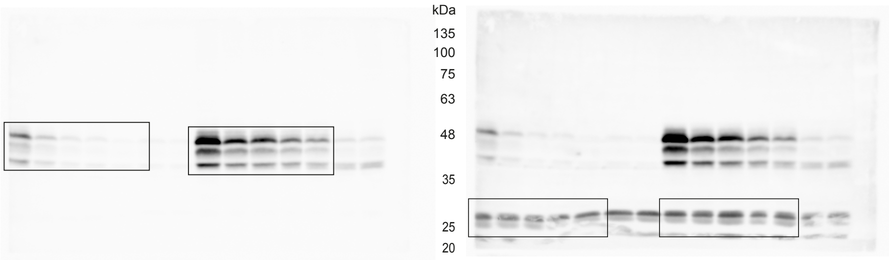

Figure 2C

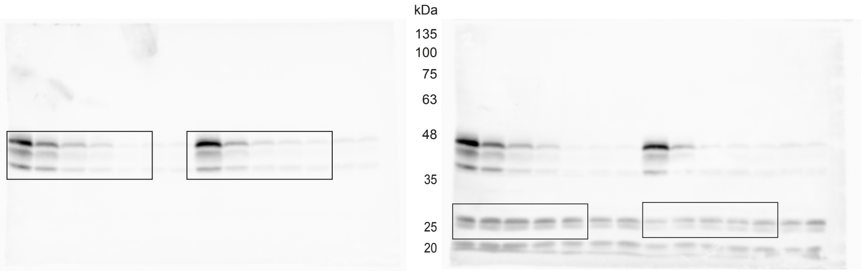

Figure 2D

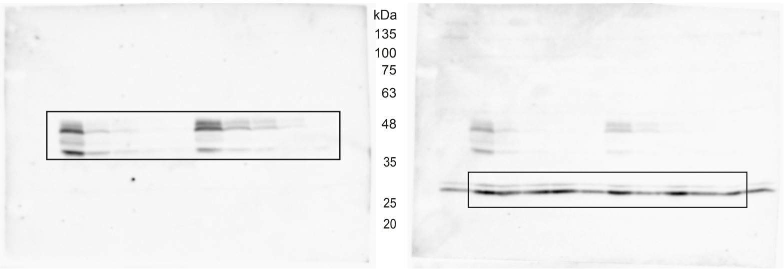

Figure 2E

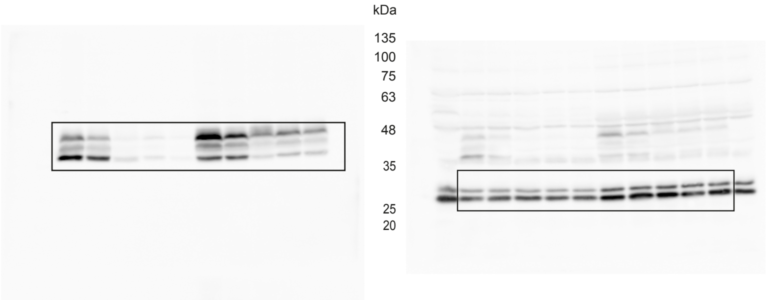

Figure 2F

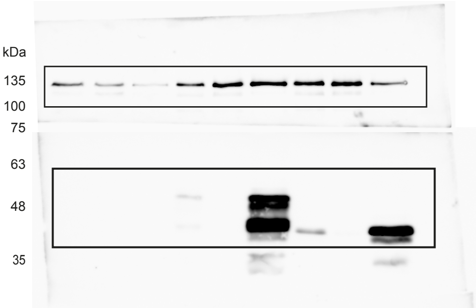

Supplementary Figure 10. Uncropped western blots shown in Figure 2

Figure 3A

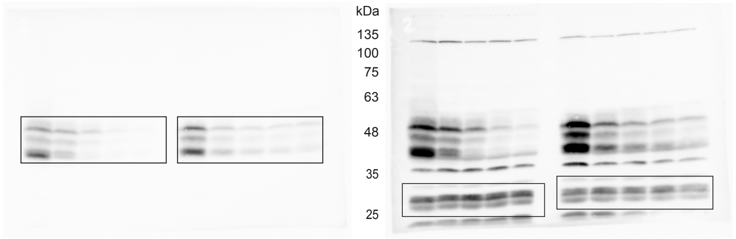

Figure 3B

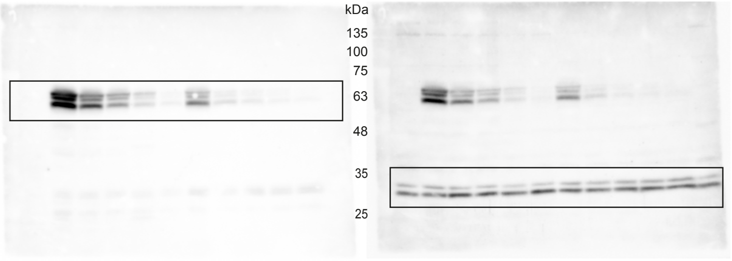

Figure 3C

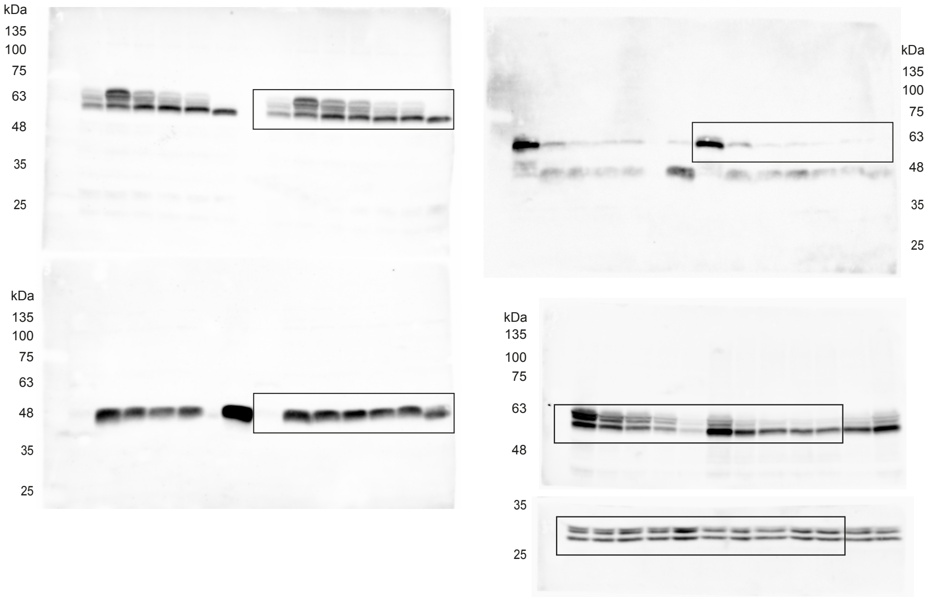

Figure 3D

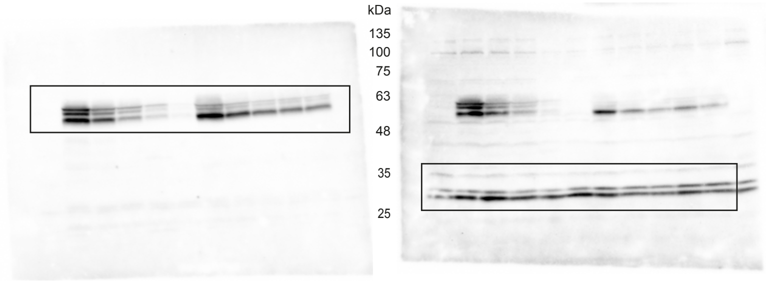

Figure 3E

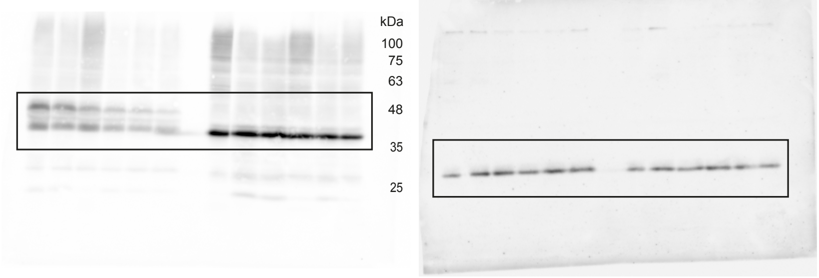

Supplementary Figure 11. Uncropped western blots shown in Figure 3

Figure 4B

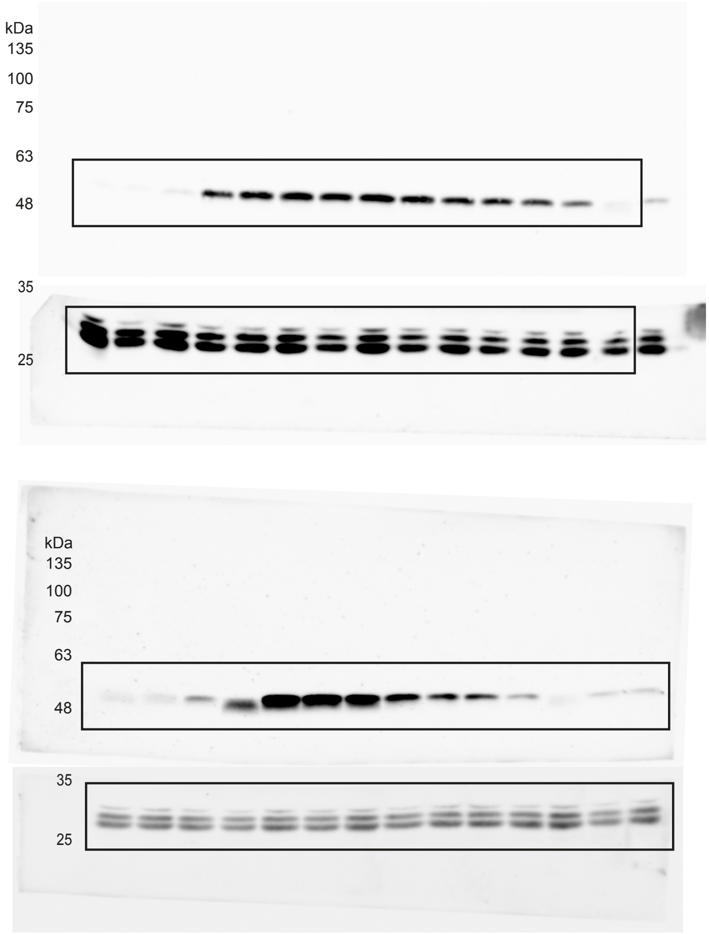

Supplementary Figure 12. Uncropped western blots shown in Figure 4

Figure 5A

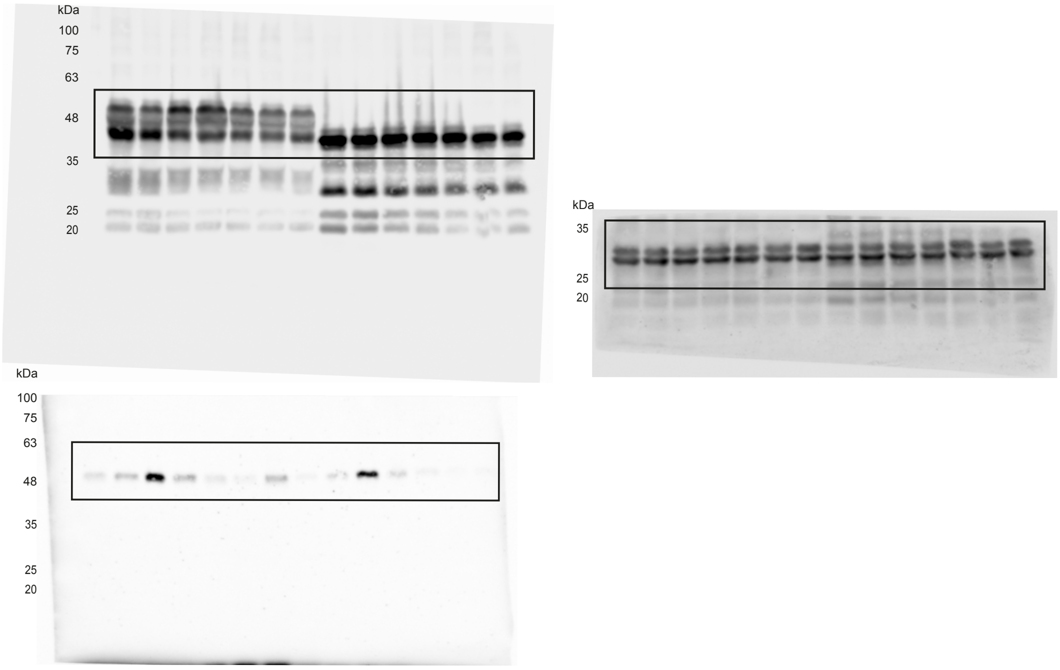

Figure 5B

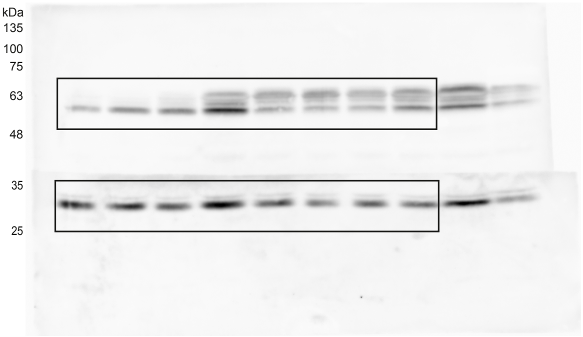

Figure 5F

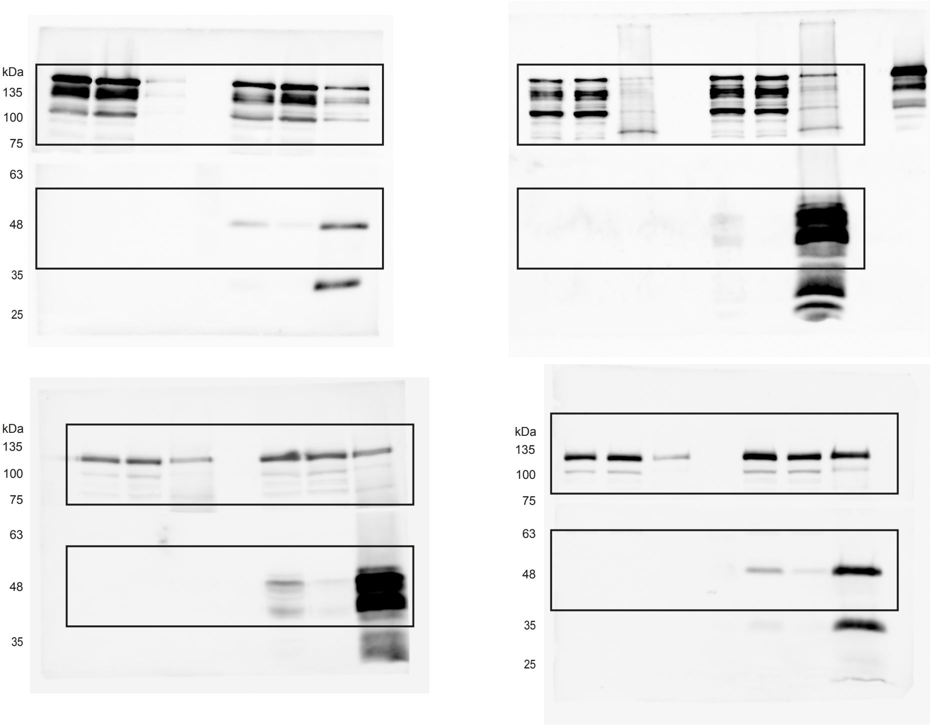

Supplementary Figure 13. Uncropped western blots shown in Figure 5

Figure 6B

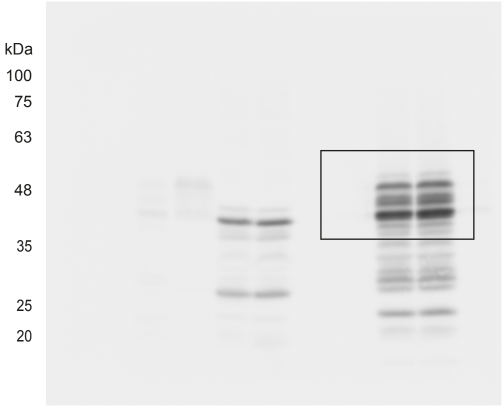

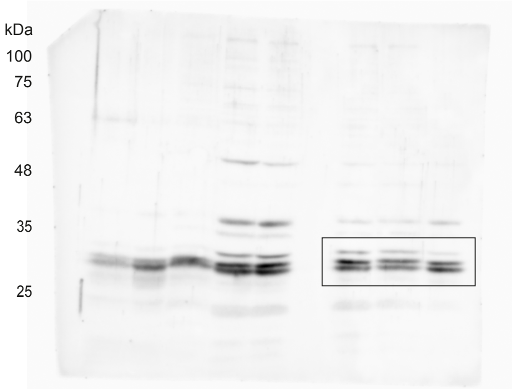

Supplementary Figure 14. Uncropped western blots shown in Figure 6

Figure 7A

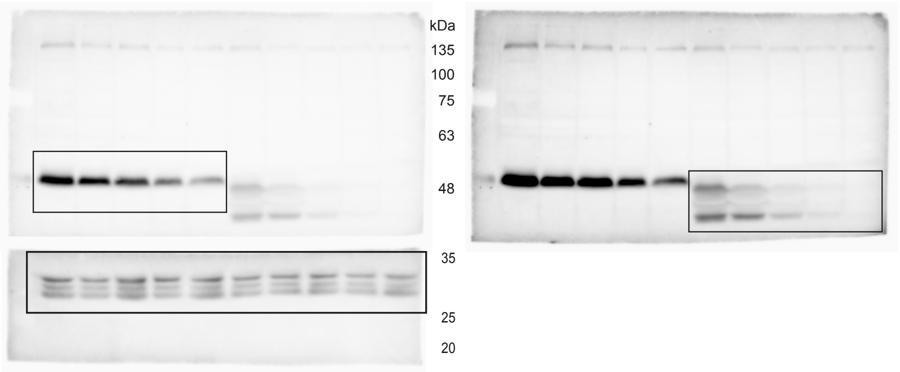

Figure 7B

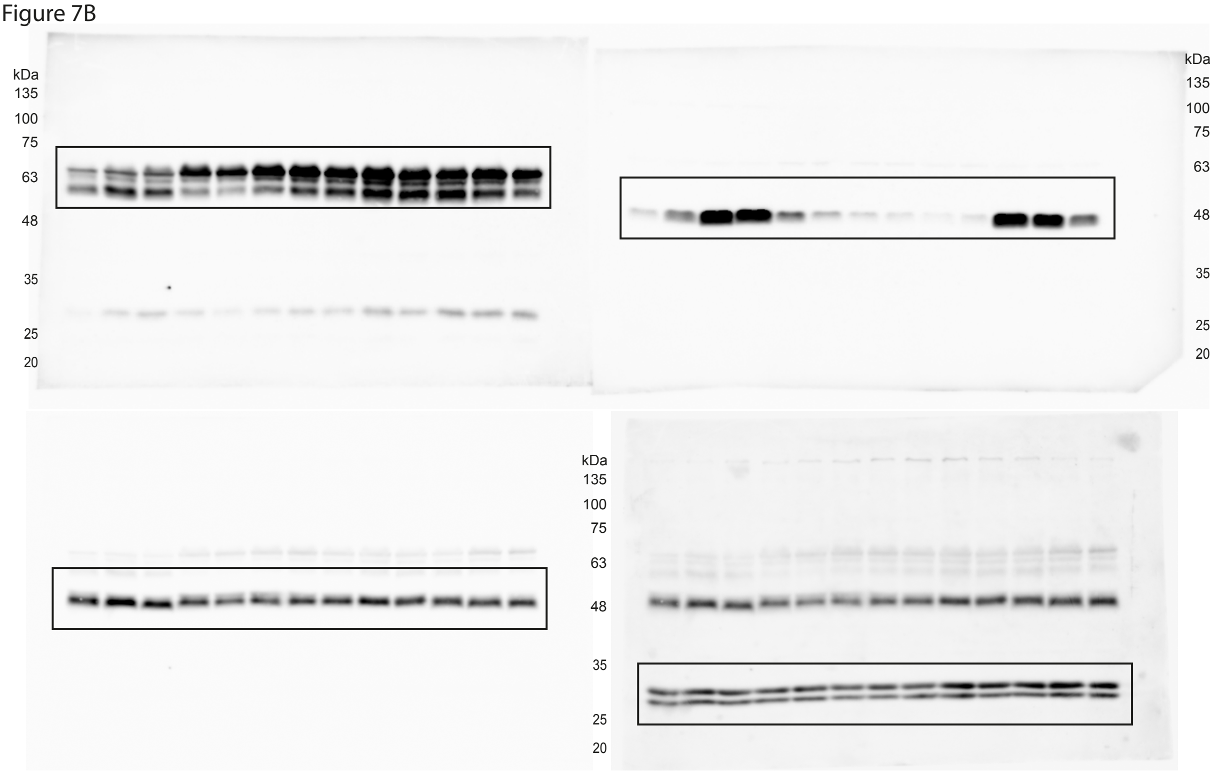

Figure 7C

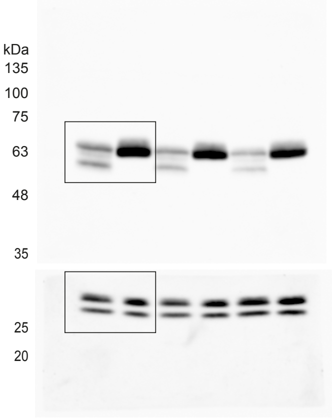

Supplementary Figure 15. Uncropped western blots shown in Figure 7

Supplementary Table 1. Yeast strains

W303-1a	*MATa ade2-1 trp1-1 leu2-3,112 his3-11,15 ura3-52 can1*
W303-1b	*MATα ade2-1 trp1-1 leu2-3,112 his3-11,15 ura3-52 can1*
WCG4α	*MATα his3-11 leu2-3,112 ura3Δ*
A364A[a]	*MATa ade1 ade2 ura1 his7 lys2 tyr1 gal1*
pre1pre2[a]	*pre1-1 pre2-2* in WCG4α
cdc4^{ts}	*cdc4-1* in W303-1a background
cdc15^{ts}	*cdc15-2* in W303-1a background
cdc16^{ts}	*cdc16-1* in A364
cdc28^{ts}	*MATa bar1 ade1 his2 leu2-3,111 trp1 ura3Δ cdc28-13*
cdc53^{ts}	*cdc53-1* in W303-1a background
BF305[c]	*GALp:CLN3 cln1::HIS3 cln2::TRP1 leu2 ura3 ade1 arg5 arg6 met14*
K3080[d]	*clb1 clb2^{ts} clb3::TRP1 clb4::HIS3* in W303-1a
MCY123	*CLB2-HA-HIS3 SIC1-MYC-TRP1* in *cdc15^{ts}*
MT244[b]	*MATa ade2-1 trp1-1 leu2-3,112 his3-11,15 ura3-52 can1 cln3::URA3*
JCY0167	*swi4::LEU2* in W303-1a
JCY0221	*swi6:: TRP1* in W303-1a
JCY0325	*swi6:: kanMX6* in W303-1a
JCY0622	*SWI6-MYC- kanMX6* in W303-1a
JCY0624	*mbp1::URA3* in W303-1a
JCY1346	*WHI5-HA-TRP1* in W303-1a
JCY1440	*cln3::TRP1* in W303-1b
JCY1539	*grr1::LEU2* in W303-1a
JCY1728	*WHI7-HA-TRP1* in W303-1a
JCY1732	*WHI7-HA-TRP1* in *cdc53^{ts}*
JCY1735	*WHI7-HA-TRP1* in A364A
JCY1737	*WHI7-HA-TRP1* in *cdc16^{ts}*
JCY1739	*WHI7-HA-HIS3* in WCG4α
JCY1740	*WHI7-HA-HIS3* in *pre1pre2*
JCY1746	*WHI7-GFP-kanMX6* in W303-1a
JCY1757	*WHI7-HA-TRP1* in *cdc4^{ts}*
JCY1760	*WHI7-HA-TRP1* in JCY1539
JCY1789	*WHI7-GFP-kanMX6* in *cdc28^{ts}*
JCY1802	*WHI7-GFP-kanMX6* in MCY123
JCY1804	*kanMX6-GAL1:WHI7* in JCY1828
JCY1815	*WHI7-GFP-kanMX6* in JCY1440

JCY1819	*whi7::KanMX6* in W303-1a
JCY1836	*SIC1-MYC-HIS3* in *cdc15^{ts}*
JCY1837	*cln3::TRP1* in JCY1836
JCY1843	*whi7::kanMX6* in JCY1836
JCY1868	*whi7::kanMX6* in MT244
JCY1872	*whi7::kanMX6* in JCY0167
JCY1874	*whi5::LEU2* in W303-1a
JCY1875	*whi5::LEU2* in MT244
JCY1879	*SWI4-myc-HIS3* in *cdc15^{ts}*
JCY1880	*whi7::kanMX6* in JCY0221
JCY1883	*kanMX6-GAL1:WHI7* in JCY1440
JCY1885	*kanMX6-GAL1:WHI7* in JCY1874
JCY1896	*whi7::KanMX6* in JCY1837
JCY1912	*WHI7-GFP-TRP1 WHI5-HA-kanMX6* in JCY1836
JCY1921	*kanMX6-GAL1:WHI7* in JCY1875
JCY1982	*SWI6-myc-HIS3* in JCY1346
JCY1984	*SWI4-myc-HIS3* in JCY1346
JCY1991	*WHI7-GFP-kanMX6* in K3080
JCY2007	*WHI5-GFP-kanMX6* in BF305
JCY2008	*WHI7-GFP-kanMX6* in BF305
JCY2009	*WHI5-GFP-kanMX6* in W303-1a
JCY2013	*Cln1::kanMX6 cln2::LEU2 in JCY1757*

[a] from Dr. H. Wolf; [b] from Dr. M. Tyers; [c] from Dr. B. Futcher; [d] from Dr. K. Nasmyth
